# The brain-gut axis of longevity

**DOI:** 10.18632/aging.103996

**Published:** 2020-09-27

**Authors:** Anubhuti Dixit, Varsha Singh

**Affiliations:** 1 Amity Institute of Neuropsychology and Neurosciences, Amity University, Noida 201313, India; 2 Department of Molecular Reproduction, Development and Genetics, Indian Institute of Science, Bangalore 560012, India

**Keywords:** longevity, aging, brain-gut axis, lipid metabolism, GPCR

The landmark study by Kenyon et al. in 1993 showing doubling of the life span of *Caenorhabditis elegans* due to altered insulin-like growth factor signalling is considered by many as the first milestone in the long-standing quest for ‘longer life’ [1]. Decrease in growth factor signalling extends life span not only in worms but also in mammals. Since then, a number of genetic factors acting at cellular, tissue and organismal levels have been shown to regulate longevity and to ameliorate aging. This includes chromatin remodelling factors, transcription factors, enzymes involved in lipid metabolism and components of the cellular translation machinery and of autophagy. The actions of many of these factors converge on lipid metabolism in the intestine or the gut of *C. elegans* [2,3]. However, it is not clear how various mechanisms for life span extension are coordinated in a multicellular organism?

**Figure 1:**
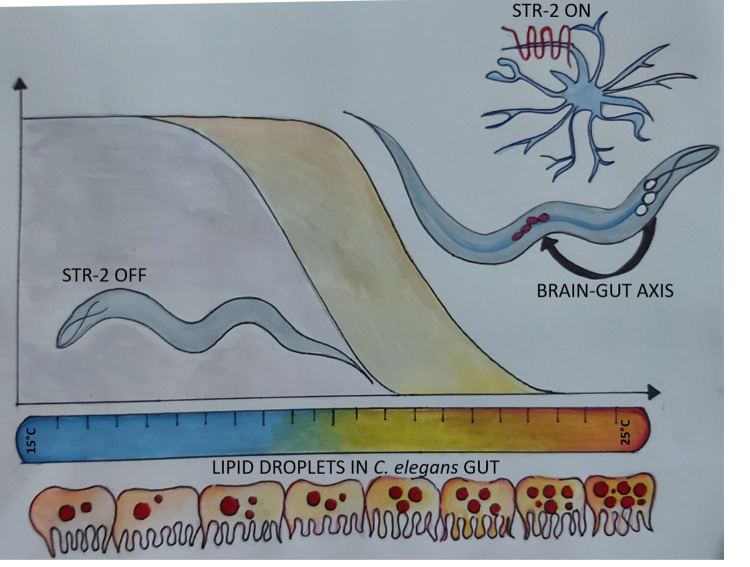
**Illustrations of the BRAIN-GUT axis of longevity operative in *C. elegans* at high temperature of growth.** STR-2, a G protein coupled receptor in three sensory neurons, regulates lipid metabolism and longevity in *C. elegans* at warmer temperature. (Artwork by Shivangi Mishra, shivangim@iisc.ac.in)

Is the brain of animals capable of coordinating and regulating longevity pathways? In 1999, an elegant study by Apfeld and Kenyon showed the role of sensory cilia in the control of life span in *C. elegans* [4]. However, the mechanism by which sensory neurons, numbering 48 in the adult nematode, exert control over aging remained elusive. Alcedo and Kenyon extended the study by systematically ablating Amphid sensory neurons [5]. Several of the ablated neurons were attributed with a role in regulation of longevity although each neuron had only a small impact. *C. elegans* sensory neurons express multiple G protein coupled receptors (GPCRs), and thus the ablation of an entire neuron may appear to have no net effect due to the absence of multiple GPCRs which may have opposing effects. Therefore, it is desirable to study individual GPCRs than entire neurons, to gain mechanistic understanding of longevity pathways. In a recent study we asked whether specific GPCRs, expressed only in the sensory neurons, exert control over tissue systems. By systematically examining the role of olfactory GPCRs in the regulation of longevity, we found that serpentine receptor STR-2 is involved in the regulation of longevity in *C. elegans* [6]. One of the surprising findings is that this olfactory GPCR, restricted to just three neurons, has a rather large impact on life span. Activity of STR-2 in olfactory neurons AWC and chemosensory neurons, ASIL and ASIR, was necessary for longevity on *E. coli* diet and at high temperature of growth above 20°C. This suggested that STR-2 is involved in sensing temperature and/or dietary cues. Indeed, a bacterial secondary metabolite 2-heptanone is a volatile ligand for STR-2 GPCR [7], nonetheless this ligand was undetectable in *E. coli* OP50, the laboratory diet of *C. elegans* on which life span assays were performed. By tracking calcium levels in AWC and ASI neurons at different temperatures, we found that STR-2 is necessary for temperature response in these neurons. Detailed metabolomic analysis showed that decreased levels of lipid droplets and monounsaturated fatty acids (MUFAs) were the contributing factors for shorter life span of the receptor deficient mutant. Indeed, *str-2* mutants had low expression of some of the key lipid metabolic enzymes such as delta-9 desaturases required for MUFA production and diacylglycerol acyltransferase required for neutral lipid synthesis. Consequently, dietary supplementation of MUFAs and increasing the neutral lipid levels by an additional 30% could restore the life span of *str-2* mutant animals. Although STR-2 activity is restricted to three neurons in the head region, its effect is mediated via the perturbation of lipid metabolism in the intestine of *C. elegans*. Thus, STR-2 regulates a brain-gut axis of longevity and metabolism (Figure 1). The future possibilities for research in this area promises to be very exciting. The question of broad interest in animal physiology is understanding and harnessing mechanisms of control of metabolism by the nervous system. Since *C. elegans* intestine is not innervated, one can investigate a neuropeptide-based mode of communication between the brain and the gut. The second question would be to investigate how and where the known regulators of metabolism and longevity such as chromatin remodelling factors integrate into the brain-gut axis. Do olfactory capabilities of worms and mammals regulate life span? Could we utilize ligands for olfactory GPCRs including those for STR-2 and food derived olfactory cues to alter metabolism in *C. elegans* and mammals? Going forward, such studies in *C. elegans* will allow us to evaluate and investigate pharmaceutical potential of dietary volatiles for the extension of organismal life span.
